# Allogenic iPSC-derived RPE cell transplants induce immune response in pigs: a pilot study

**DOI:** 10.1038/srep11791

**Published:** 2015-07-03

**Authors:** Elliott H Sohn, Chunhua Jiao, Emily Kaalberg, Cathryn Cranston, Robert F. Mullins, Edwin M. Stone, Budd A. Tucker

**Affiliations:** 1 Stephen A Wynn Institute for Vision Research, Department of Ophthalmology, University of Iowa Hospitals and Clinics, Iowa City, IA

## Abstract

Stem cell strategies focused on replacement of RPE cells for the treatment of geographic atrophy are under intense investigation. Although the eye has long been considered immune privileged, there is limited information about the immune response to transplanted cells in the subretinal space of large animals. The purpose of this study was to evaluate the survival of allogenic induced pluripotent stem cell-derived RPE cells (iPSC-RPE) delivered to the subretinal space of the pig as well as determine whether these cells induce an immune response in non-diseased eyes. GFP positive iPSC-RPE, generated from outbred domestic swine, were injected into the subretinal space of vitrectomized miniature swine. Control eyes received vehicle only. GFP positive iPSC-RPE cells were identified in the subretinal space 3 weeks after injection in 5 of 6 eyes. Accompanying GFP-negative cells positive for IgG, CD45 and macrophage markers were also identified in close proximity to the injected iPSC-RPE cells. All subretinal cells were negative for GFAP as well as cell cycle markers. We found that subretinal injection of allogenic iPSC-RPE cells into wild-type mini-pigs can induce the innate immune response. These findings suggest that immunologically matched or autologous donor cells should be considered for clinical RPE cell replacement.

Degenerated retinal pigment epithelial cells (RPE) is a unifying feature associated with central vision loss in common blinding diseases such as age-related macular degeneration^1^,^2^,^3^ and more rare, inherited macular dystrophies such as Best Disease and Stargardt Disease^4^,^5^,^6^,^7^. Though numerous studies, including clinical trials, are currently underway, no FDA-approved therapies to treat RPE loss associated with inherited retinal degenerations or geographic atrophy (GA) exist^8^. Even if prevention of GA could be achieved, this would do little to help the millions of people already blinded by this form of AMD^9^. The ability to effectively replace atrophic RPE, in addition to photoreceptors and choriocapillaris, is thus of high priority.

Ideally, proof-of-concept cell replacement strategies demonstrating lack of immune response, safety, cellular survival, integrative capacity, and retinal function would be developed in a large animal model prior to introduction into humans. With an eye that is very similar to that of the human in both size and retinal structure (i.e. 10-layered cellular retina, rod:cone ratio, a cone-rich visual streak akin to the macula) the pig is arguably the ideal large animal model for such studies^10^,^11^,^12^,^13^. In addition, several pig models of retinal degeneration, which arguably present fewer ethical concerns than non-human primates, exist^14^,^15^.

The anterior chamber of the eye is generally considered to have immune privilege through a process known as anterior chamber associated immune deviation (ACAID). ACAID is represented by a downregulation of the Th1 immune response when foreign antigens are introduced into the anterior chamber. From a cytokine perspective, ACAID represents a favorable balance of immune mediators; e.g. TGF-β downregulates Th1 response allowing foreign antigens to be better tolerated^16^. Although ACAID is often generalized to the rest of the eye, it is apparent that the subretinal space is not afforded the same degree of immune privilege as the anterior chamber, i.e. rejection of photoreceptor and RPE cells has been seen following subretinal injection^1^,^2^,^3^,^17^,^18^,^19^. Allogenic stem cell derived retinal cells, such as those generated from embryonic stem cells, are being considered for human therapy (e.g.^20^). Careful examination of the post-transplant immune response in a large animal model following injection of an allogeneic cell source is needed to determine the feasibility of this approach. To date, immunologic studies of large animal eyes involving transplantation of any retinal cell type are lacking. There are also few studies investigating the immune response to iPSC-derived cells in the eye. We sought to assess the feasibility and characterize the immune response to subretinal injection of allogenic iPSC-derived RPE cells in wild-type pigs.

## Methodology

### iPSC generation

iPSCs were generated from adult GFP positive swine fibroblasts^21^,^22^ via infection with four separate non-integrating/footprint-free Sendai viruses, each of which were designed to drive expression of one of four transcription factors: OCT4, SOX2, KLF4, and c-MYC (A1378001, Invitrogen, Grand Island, NY). Fibroblasts plated on six-well tissue culture plates were infected at an MOI of 5. At 12–16 hours post-infection, cells were washed and fed with fresh growth media (DMEM/F12 [Gibco], 10% heat inactivated FBS [Gibco] and 0.2% primocin [Invivogen]). At 7 days post-infection, cells were passaged onto 10CM dishes pre seeded with 1 million mouse embryonic fibroblasts (ATCC) at a density of 300,000 cells/well and fed every day with pluripotency media (DMEM F-12 media [Gibco], 20% knockout serum replacement [Gibco], 0.0008% beta-mercaptoethanol [Sigma-Aldrich, St. Louis, MO], 1% 100 × NEAA [Gibco], 100 ng/ml bFGF [human] [R&D], and 0.2% primocin [Invivogen]. At 3 weeks post-viral transduction, iPSC colonies were picked, passaged onto synthamax coated plates (Corning), and clonally expanded under feeder free conditions for a minimum of 10 passages prior to induction of differentiation. During reprogramming and maintenance of pluripotency, cells were cultured at 5% CO_2_, 5% O_2_, and 37 °C.

### RPE cell differentiation

To maintain pluripotency, adult-derived iPSCs were cultured in xeno/feeder free cell culture media. To initiate differentiation, iPSCs were switched from pluripotency media to RPE media (DMEM media [Gibco], containing 10% KSR [Gibco], 1% NEAA, 0.2% primocin [Invivogen]). Cultures were fed every other day for 40 days at which time pigmented clusters were isolated, dissociated and replated in fresh RPE media on synthamax coated plates (Corning). Pigmented cells were allowed to attach for 48 hours prior to changing media. Cultures were fed every other day with fresh RPE cell media until cells reached confluence. Prior to transplant, cells were washed 3 times with HBSS^-ca-mg^ , were passaged using triple express (Invitrogen), and were resuspended in HBSS^-ca-mg^ for injection.

To demonstrate cellular identity post-RPE cell generation and expansion, phase microscopy (to determine percent of pigmentation) and immunocytochemical analysis (to determine number of cells expressing pluripotency vs. RPE cell markers) was performed. For immunocytochemical analysis cells were fixed in a 4% paraformaldehyde solution and immunostained as described previously^23^,^24^. Briefly, cells were incubated overnight at 4 **°**C with antibodies targeted against either the pluripotency markers Nanog (Abcam) and SSEA1 (Thermo Fisher), the RPE cell transcription factors MITF (Abcam) and Pax6 (Millipore), or the tight junction marker Zo-1 (Millipore). Subsequently, Cy2 or Cy3-conjugated secondary antibodies were used (Jackson Immunochem, West Grove, PA) and the samples were analyzed using fluorescence microscopy. Microscopic analysis was performed such that exposure time, gain, and depth of field remained constant between experimental conditions.

### Cell Counting

Cell counts were performed by counting the total number of cells containing pigment expressing MITF, PAX6, and ZO-1. In each case counts were performed using 9 microscopic fields from each of three experimental repeats. As such, statistical analysis was based on counts from 9 microscopic fields (approximately 2500 cells in total were counted for each marker analyzed).

### Surgery and undiluted vitreous sample collection

**Figure 1:**
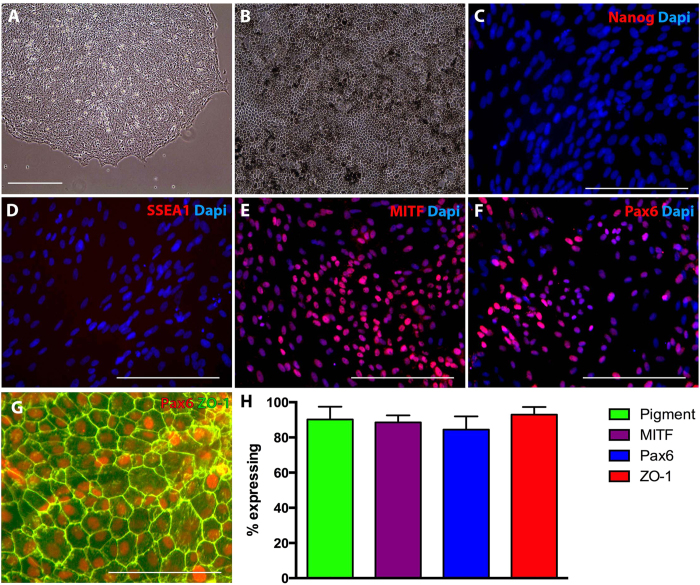
Generation of porcine iPSC derived RPE cells. **A**: Representative phase micrograph of pig iPSC colonies consisting of densely packed cells with high nucleus to cytoplasm ratio. **B**: Densely pigmented porcine iPSC-derived RPE cells with typical hexagonal morphology. **C**–**G**: Immunocytochemical analysis of porcine iPSC derived RPE cells targeted against Nanog (C), SSEA1 (D), MITF (E), Pax6 (F & G) and ZO-1 (G). **H**: Histogram depicting the total number of cells post-differentiation that were pigmented, expressed MITF, Pax6, and ZO-1.

All animal procedures were approved by the Institutional Animal Care and Use Committee of the University of Iowa and conducted in accordance with the ARVO Statement for the Use of Animals in Ophthalmic and Vision Research. 12–14 week old non-immune suppressed wild-type yucatan miniature swine (in-bred compared to domestic swine which are outbred) underwent 23 gauge pars plana vitrectomy, including induction of posterior vitreous detachment, under general anesthesia. Undiluted vitreous samples (i.e. native vitreous) were extracted at the start of vitrectomy. A 41 gauge extendible flexible polyamide cannula (DORC, New Hampshire, USA) was used to create a small retinotomy to allow a 300 microL subretinal bleb containing a bolus of 250,000 allogeneic iPS-derived green fluorescent protein-positive retinal pigment epithelial cells to be injected in the cone-rich visual streak region (Fig. 1). Sclerotomies were closed with 7-0 vicryl suture. 5% povidone-iodine was used to rinse the eye. The contralateral eye (control) underwent the same procedure described above with the exception of a bleb containing balanced salt solution (BSS) devoid of cells. Vitreous samples were taken from all eyes at post-operative week three.

### Histological Procedures and Morphology

All eyes were enucleated three weeks after surgery and processed for histological assessment. Six subretinal iPSC-RPE injected eyes and five control eyes were evaluated. Whole eyes were fixed in 4% paraformaldehyde overnight. After cornea and lens were gently removed, the eyes were cryoprotected in serial sucrose solutions^25^, and then bleb-injected regions from the eyes were dissected and embedded in OCT compound in liquid nitrogen. Transverse 10 μm cryosections were cut, mounted onto Superfrost Plus glass slides and stored at −80 °C until use.

Groups of sections were thawed and air dried at room temperature, washed with phosphate-buffered saline (pH 7.4) for 5 minutes, then stained with H&E and Masson’s trichrome for light microscopy. Adjacent sections were blocked with 2% bovine serum albumin for 1 hour, and then incubated with the following primary antibodies for immunohistochemical assessment: tight junction marker ZO-1 (Millipore, dilution 1:200), RPE marker BEST1 (Abcam, dilution 1:200), anti-macrophage (CD68; AbD Serotec, dilution 1:1000), anti-CD45 (AbD Serotec, dilution 1:200), anti-IgG-Fc fragment (Bethyl lab, Inc., dilution 1:200), anti-GFAP for astrocyte/Muller cell marker (Neomarker, dilution 1:100), anti-Ki67 for cellular proliferation marker (Dako, M7240, dilution 1:200), anti-PCNA for proliferating cell nuclear marker (Abcam, ab29), and anti-Nestin for neural stem cell marker (Millipore, dilution 1:200). After primary antibody incubation over night at 4 °C, sections were probed with appropriate secondary fluorescence antibody (Jackson ImmunoResearch Lab) for 1 hour at room temperature, and then mounted with anti-fade medium with DAPI. Negative controls for immunohistochemistry were performed in parallel by omission of primary antibodies. All sections were analyzed and imaged with an Olympus BX41 fluorescence microscope. Additional porcine tissues were collected and preserved as described above in order to have positive controls for each of the utilized antibodies (Table 1).

### Quantibody Cytokine Array and Statistical Analysis

All vitreous samples were centrifuged at 12,000 rpm at 4 °C for 15 minutes, then the supernatant snap frozen in liquid nitrogen for storage. Vitreous was assayed for cytokine levels using a swine cytokine Quantibody array kit (RayBiotech, Inc.) according to the manufacturer’s instructions. Data were analyzed by Students t-test to compare two groups and one-way ANOVA followed by Fisher’s LSD test to compare three groups by using SPSS software. Bonferroni-adjusted p-values were used for multiple comparisons. Results are expressed as mean ± SEM. P < 0.05 was considered statistical significant.

## Results

### Generation of GFP-positive iPSC-derived RPE cells

As indicated in the methods section above, iPSCs were generated from GFP positive pig fibroblasts via sendai viral transduction of the transcription factors OCT4, SOX2, KLF4, and c-MYC. At 3–4 weeks post-transduction iPSC colonies were manually isolated and expanded under feeder free conditions. As shown in Fig. 1, pig iPSC colonies consisting of densely packed cells with a high nucleus to cytoplasm ratio were obtained. Following expansion, pluripotency media was replaced with RPE cell differentiation media supplemented with 10 mM of nicotinamide. At 30–40 days post-differentiation, pigmented clusters of cells began to appear. Following isolation, dissociation and expansion of these pigmented clusters on fresh synthamax coated cell culture dishes, densely pigmented RPE cells with typical hexagonal morphology appeared (Fig. 1). Immunocytochemical analysis, using antibodies targeted against the pluripotency markers Nanog (Fig. 1C) and SSEA1 (Fig. 1D) revealed their complete lack of expression in differentiated cultures. However, cells were found to be positive for the RPE cell transcription factors MITF (Fig. 1E) and Pax6 (Fig. 1F) and the tight junction marker ZO-1 (Fig. 1G). Cell counts revealed that post-differentiation an average of 91% of the cells were pigmented, 89% expressed MITF, 84% expressed Pax6 and 93% formed tight junctions.

### Identification of GFP-positive iPSC-derived RPE cells in subretinal space

**Figure 2:**
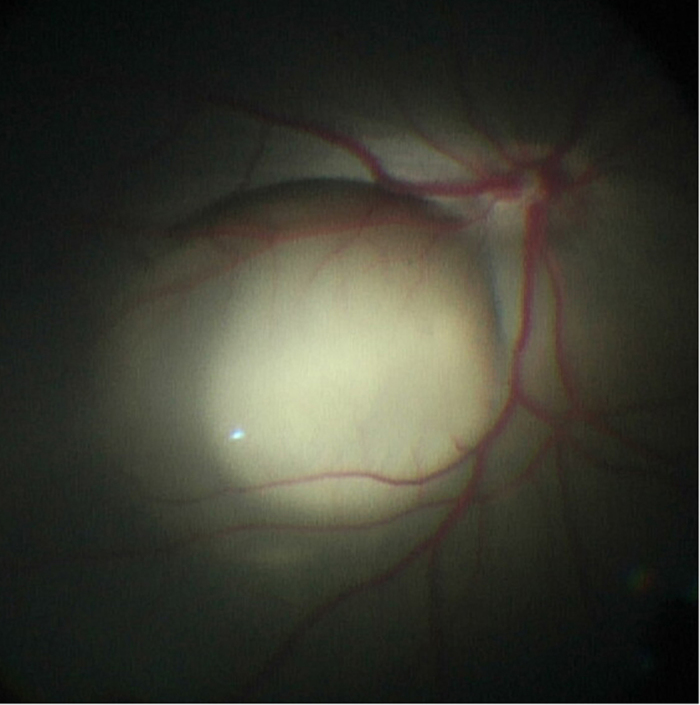
Surgeon’s view (intra-operative) of pig fundus post-vitrectomy with subretinal bleb of GFP+ iPSC-derived RPE cells in the visual streak.

**Figure 3:**
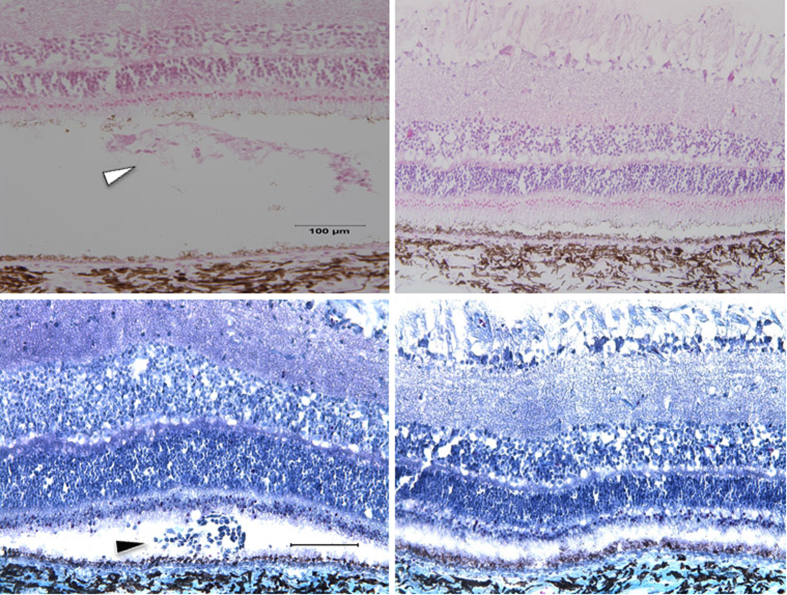
H&E (top panels) and Masson trichrome (bottom panels) stains of treated eyes (left panels) showing cellular mass and material in subretinal space (arrowheads) that was absent in the relatively normal BSS-treated control eyes (right panels). Scale bar = 100 um.

As described in the methods section above, GFP-positive iPSC-RPE cells were harvested and transplanted as a single cell suspension (250,000 cells/eye) into the subretinal space of wild type mini swine (n = 6 eyes) via 3-port vitrectomy and subretinal injection (Fig. 2). At 3 weeks post-injection of iPSC-RPE, H&E staining consistently revealed a mass of cellular material in the subretinal space, a fraction of which were positive for GFP and DAPI (Fig. 3). The remaining DAPI-positive cells were negative on the Masson trichrome stain; these cell masses were absent in all control eyes (n = 5 eyes) which displayed normal anatomy in the areas that were injected with BSS alone (Fig. 3). The retina, RPE and non-injected areas were otherwise normal.

**Figure 4:**
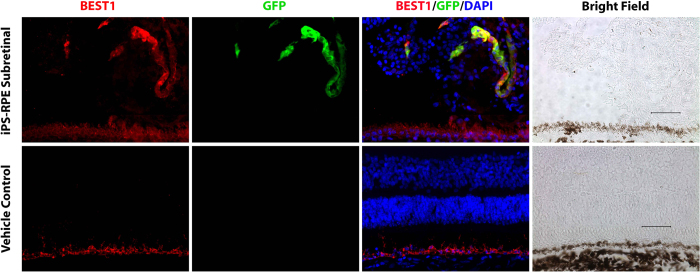
Top panels, co-immunolabeling studies using GFP and Best1 demonstrating identification of injected GFP+ iPS-derived RPE cells in the subretinal space accompanied by a large cluster of GFP-negative cells. Below panels, normal BSS-treated control eye. Scale bar = 100 um.

**Figure 5:**
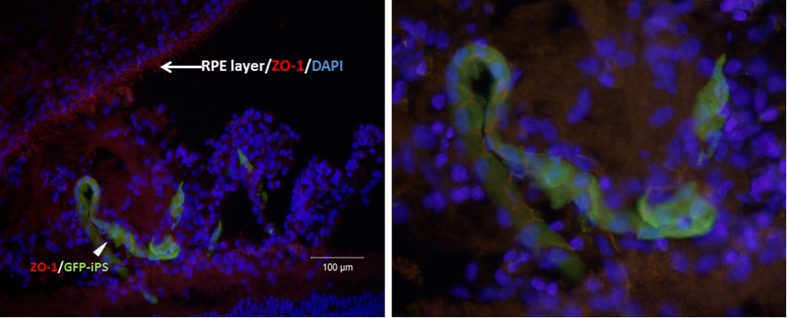
Left panel, immunolabeling of subretinal space in treated eye showing unperturbed native RPE (cell borders expressing ZO-1 marker in red) in the upper left and GFP+ iPSC-derived RPE cells forming a single chain in the subretinal space. Right panel, higher magnified view of the GFP+ iPSC-derived RPE cells expressing ZO-1 marker. Note accompanying non-GFP DAPI positive cells in the subretinal space. Scale bar = 100 um.

Co-labeling studies revealed that five of the six eyes injected with iPSC-RPE expressed GFP-positive cells in the subretinal space. These GFP-positive cells were positive for Best1 and ZO-1, indicating that these were the RPE cells that were originally injected. Only one of the six iPSC-RPE injected eyes had GFP-positive cells in a single chain of cells that could be recognized as a monolayer but did not have the typical polarized organization of the native RPE. Integration into the wild-type unperturbed RPE was not observed in any eyes (Figs 4 and 5). No GFP positive cells were observed in the control BSS-injected eyes (Fig. 4).

### Immune response in the subretinal space

**Figure 6:**
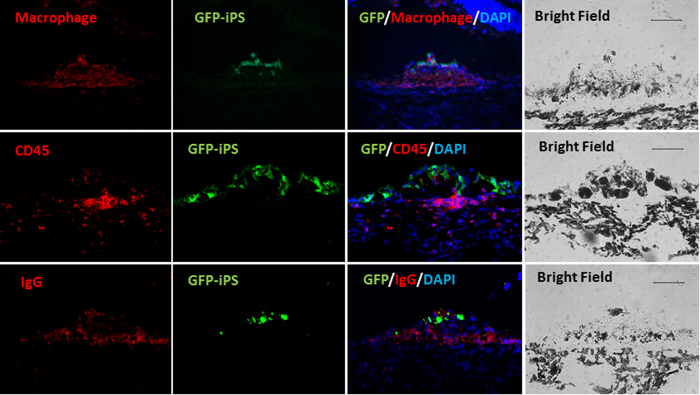
Immunolabeling of iPSC-RPE bolus injections in the subretinal space where the GFP-iPSC panels indicate transplanted cells (labeled in green). The merged panels demonstrate presence of accompanying GFP-negative, non-iPSC-derived cells that are positive for macrophage (top panels), CD45 markers (middle panels), and IgG (lowest panels). Corresponding bright field panels are on the far right column. Scale bar = 100 um.

A significant portion of the non-GFP DAPI stained cells accompanying the iPSC-derived RPE cells were positive for the anti-macrophage marker targeted against CD68, and to a lesser extent, the leukocyte marker CD45 (Fig. 6)^9^,^26^,^27^. No evidence of the cell cycle markers Nestin, Ki67, or PCNA were found. GFAP antibody labeling was similarly negative. Positive control tissues showed immunoreactivity for the studied antibodies. Table 1 summarizes the results of these immunofluorescence labeling studies.

### Vitreous cytokine studies demonstrating immune modulation to subretinal injection of iPSC-RPE cells

**Figure 7:**
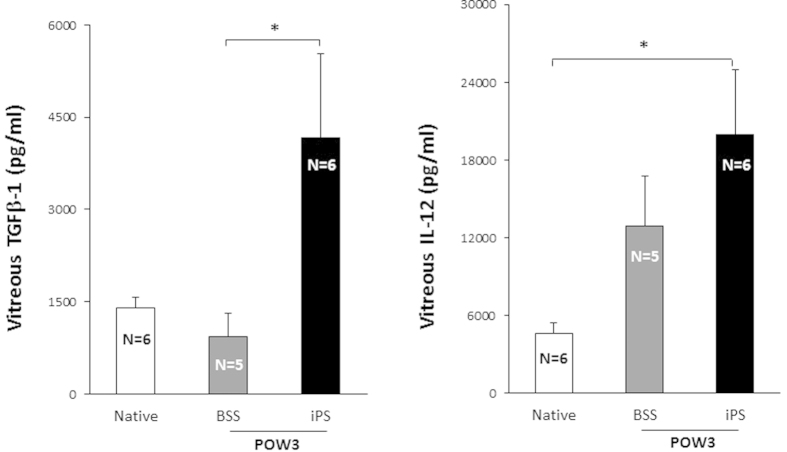
Left, Vitreous TGFβ-1 levels were higher in the iPSC-treated eyes compared to BSS-treated controls (did not reach significance compared to undiluted vitreous). Right, Vitreous IL-12 levels were higher compared to undiluted vitreous (did not reach significance compared to BSS controls). Results are expressed as mean ± SEM. * indicates P < 0.05 which was considered statistical significant. POW3 = post-operative week 3.

Vitreous TGFβ-1 levels in the treated group (4164 pg/ml) were significantly higher than BSS-injected controls (928 pg/ml; p value = 0.027); while higher compared to the native, undiluted vitreous samples (1405 pg/ml), this did not reach statistical significance (p value = 0.06). Vitreous IL-12 levels were higher in the treated group (19963 pg/ml) compared to the native, undiluted vitreous (4650 pg/ml, p value = 0.032) but not significantly higher than the BSS controls (12878 pg/ml, p value = 0.33) (Fig. 7). Cytokines IL-1β IL-4, IL-6, IL-8, IL-10, IFN-γ GM-CSF, and TNF-α were not significantly different between the treated group and either the BSS or native vitreous samples.

## Discussion

This study demonstrates that iPSC-RPE cells can persist in the subretinal space of wild type pigs three weeks after bolus cell injection without evidence of tumor formation. Integration of these cells were not observed in the host RPE—unsurprising considering this was a non-diseased, wild-type animal. However, a positive cellular immune response was observed, suggesting relative intolerance of the host to the allogenic transplant. Elevated IL-12 levels in the vitreous of eyes injected with iPSC-RPE cells compared to undiluted vitreous suggest the possibility of early activation of a T-cell-based immune response^10^,^11^,^12^,^13^,^14^,^15^,^28^,^29^.

The immune response seen in this study is plausible as the foreign antigen (i.e. allogenic iPSC-RPE cells) likely resulted in the production of an IgG antibody response demonstrated in the subretinal space. It is already well established that activated macrophages produce IL-12 which in turn play an important role in T cell response^16^,^28^,^29^,^30^. Activation of macrophages in the subretinal space was reflected by the elevated IL-12^29^ seen in vitreous samples of treated eyes. The presence of CD45+ leukocytes provides further evidence of T cell activation^26^ in the subretinal space. Nevertheless, an upregulation of the cytokine TGF-β in the vitreous, which was potentially an attempt to down-regulate the Th1 immune response^16^, was not sufficient to overcome the otherwise robust immune activation. As these animals were not immune suppressed, it is unclear whether systemic or even local steroids administered in the perioperative period could ablate the transplant rejection seen here^31^.

Since the control eyes did not receive injections of any type of iPS-derived cell (e.g. autologous), we cannot completely exclude the possibility that a reagent used to generate iPSCs like fetal bovine serum, could have caused the immune response. It is also worth noting that the injected cells were derived from an outbred domestic swine and injected into in-bred yucatan mini swine, thus could also be a cause of the immune response. Nevertheless, it is unlikely that the retinal detachment, other reagents such as balanced salt solution, or surgery itself would cause this response as there was no abnormal cellular material seen in the controls that received iatrogenic retinal detachments containing balanced salt solution alone. Taken together, the combination of histopathologic data along with elevated cytokine levels in vitreous suggests that allogenic iPSC-RPE cells induce the innate immune response in the subretinal space.

Different strategies for RPE cell replacement have been attempted with varied success. Autologous RPE translocation^32^,^33^,^34^,^35^ is an attractive surgical strategy as the immune response is expected to be minimal, however some eyes in early human trials have experienced retinal detachment, proliferative vitreoretinopathy, and/or hemorrhage. Fetal retinal-RPE sheets have also been transplanted into humans with no definite immune response but tissue was not examined to completely exclude graft rejection^36^,^37^,^38^,^39^,^40^. Ethical concerns and a need for chronic immune suppression are similarly raised issues with RPE cells derived from human embryonic stem cells^41^,^42^. Patient-specific (i.e. autologous) iPSC derived RPE cells represent an attractive cell source where the host may not need extensive immune suppression as transplanted cells would be patient matched. Derivation of RPE cells from iPSCs have already been successfully completed in both animals and humans^43^,^44^,^45^,^46^,^47^. Clinical trials focusing on the use of autologous iPSC-RPE are currently underway. Likewise, HLA matched iPSCs, so-called super donors, and the RPE derived from these cells, are also being actively investigated. Although the degree to which immunological matching required between host and donor for successful transplantation remains to be determined, these studies suggest that successful allogenic transplantation may require some degree of immune suppression. This may be especially true in AMD, in which elevated immune-mediated processes are a major component of pathology (e.g.^48^,^49^).

This is the first report to our knowledge demonstrating survival of RPE in the subretinal space derived from iPSCs in a large animal model. Whether these cells would integrate into an animal with diseased RPE remains to be seen, and it is further believed by some that a scaffold^50^,^51^,^52^,^53^ will eventually be needed to support cell polarization and integration into the host. While these studies are on-going, we demonstrated that the subretinal space does not appear to have the same immune privilege afforded to the anterior chamber. The activated adaptive immune response generated from allogenic cells suggests the need to consider use of autologous iPSC-derived cells or immune-matched cells in the subretinal space to obviate chronic immune suppression.

## Additional Information

**How to cite this article**: Sohn, E. H. *et al.* Allogenic iPSC-derived RPE cell transplants induce immune response in pigs: a pilot study. *Sci. Rep.* **5**, 11791; doi: 10.1038/srep11791 (2015).

## Figures and Tables

**Table 1:** Identification of cell mass in pig subretinal space.

Antibody	Cell Specifity	Test results in cell mass	Test results in pig tissue (4% FPA)				
			Brain	Liver	Lung	Spleen	Heart
Nestin	Neural stem cell marker	−	−	−	−	+++	−
Ki67	Cellular proliferation marker	−	+	++	+++	++++	++
PCNA	Proliferating cell nuclear	−	−	−	−	+++	−
CD45	Monocyte, leukocyte	+	−	−	−	+++	−
Macrophage	CD68	+	−	−	−	+++	−
GFAP	Astrocyte	−	++	−	−	−	−
IgG	IgG-Fc Fragment	+	NT	NT	NT	++++	NT

NT =not tested. The intensity of positive labeling was assessed as + to ++++. − mean no immunolabeling seen.
